# Electrochemical Properties of Nanoporous Carbon Material in Aqueous Electrolytes

**DOI:** 10.1186/s11671-016-1241-z

**Published:** 2016-01-13

**Authors:** Bogdan I. Rachiy, Ivan M. Budzulyak, Vitalii M. Vashchynsky, Nataliia Ya. Ivanichok, Marian O. Nykoliuk

**Affiliations:** Vasyl Stefanyk PreCarpathian National University, 57 Shevchenko Str., Ivano-Frankivsk, 76018 Ukraine

**Keywords:** Nanoporous carbon material, Aqueous electrolyte, Capacity, Voltage drop, 81.05.Uw, 82.45.Gj, 82.47.Uv, 88.80.Fh

## Abstract

The paper is devoted to the study of the behavior of capacitor type electrochemical system in the К^+^-containing aqueous electrolytes. Nanoporous carbon material (NCM) was used as the electrode material, obtained by carbonization of plant raw materials with the following chemical activation. Optimization of pore size distribution was carried out by chemical-thermal method using potassium hydroxide as activator. It is shown that obtained materials have high values of capacitance which is realized by charge storage on the electrical double layer and by pseudocapacitive ion storage on the surface of the material. It is established that based on NCM, electrochemical capacitors are stable in all range of current density and material capacity essentially depends on appropriate choice of electrolyte.

## Background

Electrochemical capacitors (EC) [1–3], or so-called supercapacitors, have high power density, continuous cycle lifetimes (~10^4^), and high energy storage compared to traditional capacitors and batteries being perspective power sources. EC is used in systems which need short-term supply of current with high energy, for example, in electrical vehicles during their run in elements which interact with solar batteries to enhance their power in systems of backup power [4]. Energy storage combines electrostatic interaction between charged plates and ions from electrolyte solution and processes connected with redox reactions of electrode material in supercapacitors. As a rule, activated carbon with high surface area and metal oxides or conductive polymers that have additional Faraday type of interaction, called pseudocapacity, are used as electrode material in electrical double layer capacitors. The use of materials with high surface area and porous structure enhances efficient operation of electrode’s surface and allows getting high values of capacitance. High surface area, geometry, and pore size are factors affecting the value of capacitance. Also, surface wettability by electrolyte and presence of electro-active molecules on it are essential to the electrode material [5]. Nowadays researches are conducted for getting new electrode materials and electrolytes compatible with them for production of EC that are characterized by high capacity, stable cycle lifetimes, and low cost. Capacitance calculated for one electrode is proportional to the surface of interface of electrode/electrolyte (*S*), according to the equation *C = εε*_0_*S/d*, where *ε*_0_ is the dielectric constant, *d* is thickness of electrical double layer (EDL), and *S* is surface area of the carbon material. Thickness of EDL depends on the concentration and amount of electrolyte ions. As a rule, the amount of EDL is 20–100 nm for concentrated electrolytes. According to the previous equation, the rise of electrode’s surface area should cause the rise of capacity of EC. But there is no direct connectivity between area of nanoporous carbon material (NCM) and the capacity of EC, because some micropores are not available for electrolyte and they do not take part in the formation of EDL. It is assumed that pores which have bigger size than ions with solvation shell are better to reduce the constant of relaxation (which describes the minimum time required for getting energy which is accumulated in a capacitor with effectiveness more than 50 %) and to increase the value of capacitance of EC. The use of porous electrode materials with appropriate pore distribution for selected electrolyte can enhance electrochemical specifications of a capacitor. Properly selected pore distribution has bigger effect on the capacity than effective surface area does [6, 7]. A significant increase in capacity is observed for carbon with pores of <1 nm, where the pore size is close to the size of ions. In addition, the limitations of power connected with adsorption of cations and anions of large sizes can be minimized by the addition of ions with smaller sizes [8]. Pores of carbon material are available when their sizes correspond to the real size of ions of electrolyte.

Aqueous solutions of acids, alkali, and inorganic salt solutions of organic salts are used as electrolytes in electrochemical capacitors. It should be noted that the capacity of EC mainly depends on the capacity which is provided by the electrode material and is expressed in F/g while voltage and resistance of that device depend on the electrolyte. In this case, it would be interesting to investigate the electrochemical properties of a porous carbon material in К^+^-containing aqueous electrolytes and to evaluate the impact of these electrolytes on the capacity of EC formed on their basis.

## Methods

NCM was used as an active material, obtained from plant raw materials through carbonization with the following chemical activation. The feedstock was dry apricot seeds milled to a fraction size of 0.25–1 mm, and their carbonization was carried out in the closed furnace at 400–420 °С with a heating rate of 10 °С m^−1^. The resulting carbonated carbon was milled to a fraction size of 200–250 μm and was mixed with potassium hydroxide and water in the weight ratio *X*_k_ *=* 1, where *X*_k_ = *m*(*C*)/*m*(KOH).

The resulting mixture was observantly stirred over 1–2 h; thereafter, it was dried in the thermostat to the constant weight at 90 °С. The dried material was placed in an oven and heated in argon atmosphere to 850–920 °С with a heating rate of 10 °С m^−1^, and it was kept at this temperature for 20 min. After cooling, the resulting material was washed up in 5 % HCl aqueous solution to neutral pH and dried at 90 °С up to constant weight.

Specifications of porous structure (it means surface area and general extent) of NCM were determined on the basis of analysis of adsorption/desorption isotherms of nitrogen at the temperature of its ebullition (−196 °С), received from Quantachrome Autosorb Nova 2200e. Before measurement, the samples were degasified at 180 °С for 18 h. The specific surface area (*S*_BET_, m^2^ g^−1^) was determined by multipoint Brunauer-Emmett-Teller (BET) method in the region of the isotherm, which is limited by the range of relative pressure *P*/*P*_0_ = 0.050–0.035. The total volume of pores (*V*_total_, sm^3^ g^−1^) was calculated by the number of adsorbed nitrogen at P/P_0_ ≈ 1. The volume of micropores (*V*_micro_, sm^3^ g^−1^) and the values of surface areas of micro (*S*_micro_, m^2^ g^−1^)- and mesopores (*S*_meso_, m^2^ g^−1^) were researched by the use of t-method and density functional theory (DFT) [9].

Research on the structure was done using scanning electron microscope JSM-6700F with energodispersive system for microanalysis JED-2300. For scanning electron microscopy and X-ray microanalysis, the samples were erected on a piece of epoxy resin as a thin layer. The size of the samples did not exceed *D* = 25 mm and height 10 mm.

The electrodes of the investigated EC were formed in the form of lamel from the mixture of <NCM>:<CA>=<75>:<25>, where CA is conductive additive (graphite KS-15 of company Lonza). The resulting symmetrical electrodes were seeped by electrolyte, were separated by sealant, and placed into a two-electrode cell with a typical size of “2525,” whereafter it was sealed. The 10 % salt aqueous solution of K_2_SO_4_, 15 % KNO_3_, 20 % KCl, 25 % KOH, and 40 % KI were used as electrolytes.

The research on electrochemical properties of EC were done by galvanostatic and potentiodynamic cycling. The measurement was made using the complex AUTOLAB PGSTAT12 of the company “ECO CHEMIE” (Netherlands) and stocked with firmware GPES.

The galvanostatic measurement was made at a voltage range of 0–1 V, and current of charge/discharge was changed from 10 to 100 mA. The capacitance was calculated by the equation *C* = 2*I · t*_d_ / [(*U*_m_−*ΔU*) *· m*], where *I* is current of charge/discharge, *t*_d_ is time of discharge, *U*_m_ is maximum voltage, *ΔU* is voltage drop of short circuit of discharge circuit, and *m* is weight. The internal resistance was calculated after ten cycles of charge/discharge: *ΔU* = 2*IR.*

## Results and Discussion

**Fig. 1:**
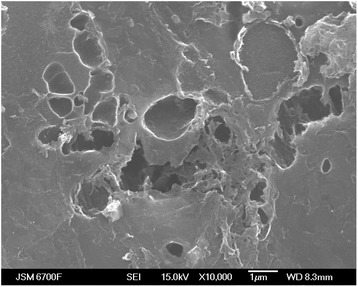
The microstructure of the NCM surface

When we study the properties of NCM, the important factor is the structure (shape, size, type) of micro- and nanopores, formed in these materials owing to technological operations at their getting and further processing. The analysis of structure by electron microscopy adds and confirms data which is gotten by low temperature porosity measurement (Fig. 1).

**Fig. 2:**
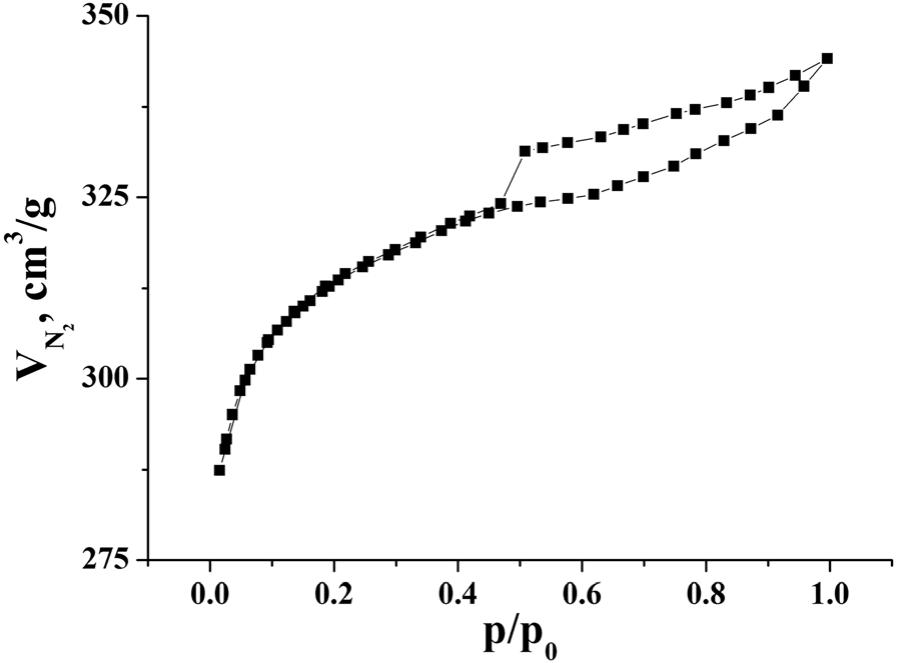
Isothermal curve of nitrogen sorption for NCM

Isotherm was gotten for NCM (Fig. 2). It is characterized for microporous materials with availability of mesopores. The hysteresis loop of the H3 type by IUPAC classification [9] is shown in the isothermal curve. This type of isothermal curve is associated with sorption processes in the micropores and capillary condensation in the meso- and macropores of organic materials and adsorption on the outer surface of the particles [10, 11].

**Fig. 3:**
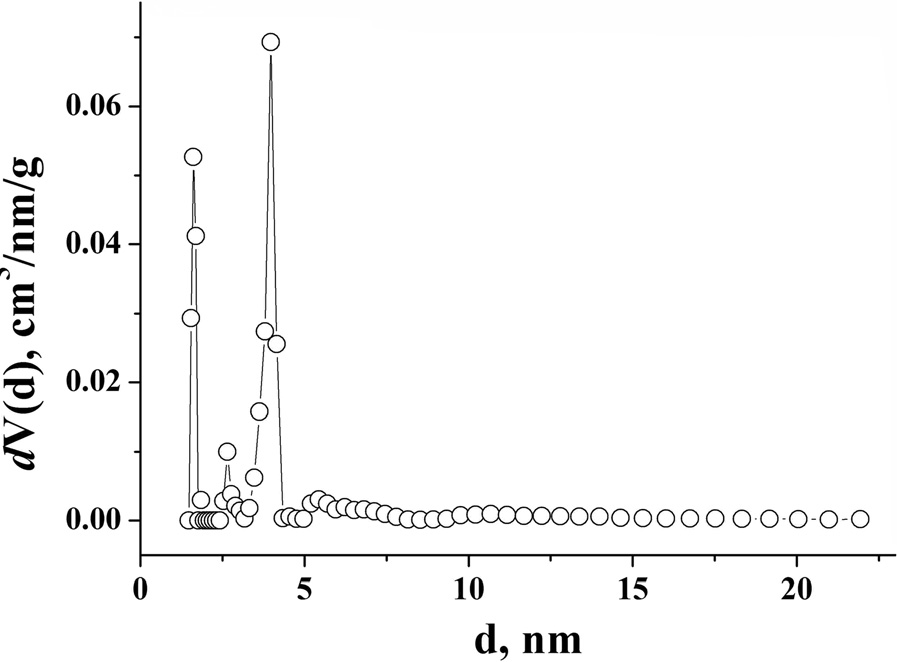
Pore size distribution for NCM (DFT method)

BET method was used for the calculation of surface area. The main properties of NCM nanostructure are shown in Table 1. Pore size distribution of carbon material was evaluated by DFT (Fig. 3). In Table 1 and in Fig. 3, it is shown that NCM has a large number of micropores (1110 m^2^g^−1^), which significantly impacts on surface area. The volume of mesopores is 15 % of pores’ total volume.

**Table 1:** Structural-adsorptional specifications of NCM

Parameter	NCM
Surface area multipoint BET, m^2^ g^−1^	1187
Total volume of pores, cm^3^ g^−1^	0.521
Volume of micropores, cm^3^ g^−1^	0.452
Surface area of micropores, m^2^ g^−1^	1110
Average diameter of pores, nm	1.76

Capacitance specifications were calculated by these methods: cycle voltammetry and galvanostatic charge/discharge [7, 12, 13].

**Fig. 4:**
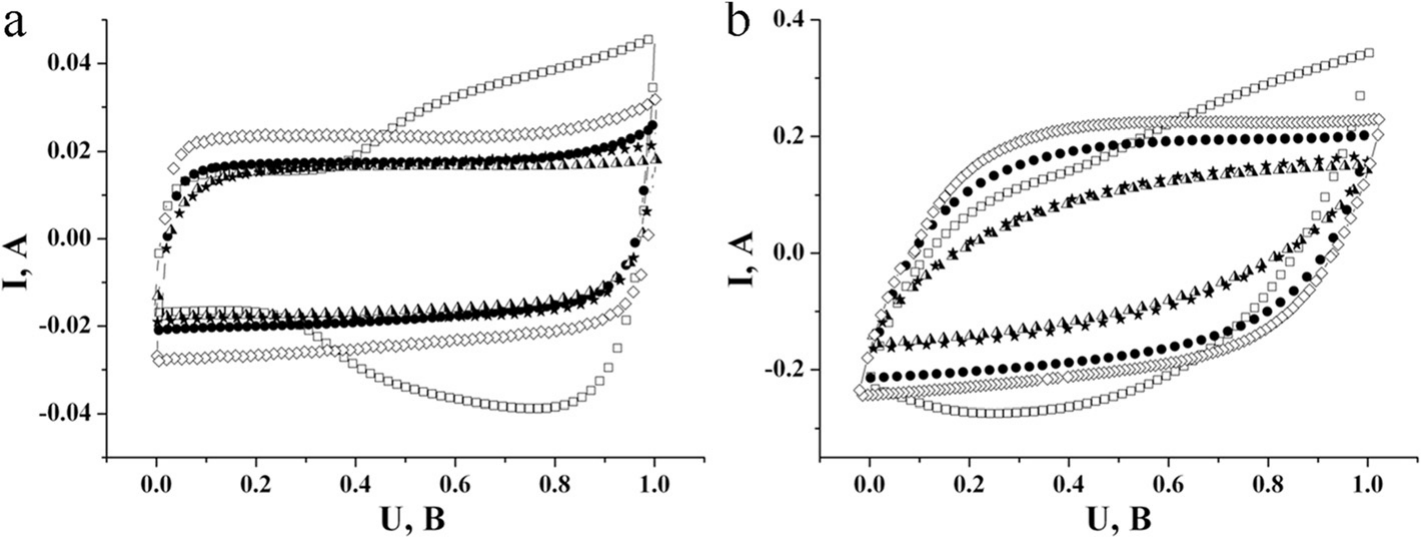
Cyclic voltammograms for NCM in К^+^-containing aqueous electrolytes at scan rates **a** 2 mV/s, **b** 20 mV/s. 40 % KI (*empty squares*), 25 % KOH (*filled circles*), 20 % KCl (*half-shaded triangles*), 15 % KNO_3_ (*filled stars*), 10 % K_2_SO_4_ (*diamonds*)

For the purpose of the determination of possible chemical reactions, electrochemical investigations were done in the range of 0–1 V for NCM in the 10 % salt aqueous solution of K_2_SO_4_, 15 % KNO_3_, 20 % KCl, 25 % KOH, and 40 % KI. In Fig. 4, cyclic voltammograms were gotten with a scan rate of 2 and 20 mV/s. At a scan rate of 2 mV/s, all CV-curves in all electrolytes, except 40 % KI, show symmetric, close to rectangular form without noticeable redox peaks, which is typical for capacity behavior. As regards the research on NCM in 40 % KI, according to the form of gotten cyclic voltammograms (Fig. 4a), we can say that before achieving a certain potential—the potential of release of Faraday transfer iodine through the electrode-electrolyte interface—the current value and capacity do not depend on the potential, which is meant to be an ideal supercapacitor. In this case, capacity is provided by the capacity of EDL, created by ions К^+^ on NCM surface. At potential, which exceeds release potential, Faraday reactions begin to store iodine on the NCM surface.

The obtained values of capacity do not show significant differences in electrolytes 10 % K_2_SO_4_, 15 % KNO_3_, 20 % KCl, and 25 % KOH at a low scan rate. At a high scan rate, they show the deviation of voltammograms from an ideal rectangular form because the transfer time of solvated ions decreased through working pores (Fig. 4b).

**Fig. 5:**
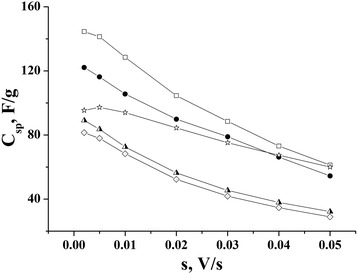
The dependence of NCM capacity on scan rate 20 mV/s: 40 % KI (*empty squares*), 25 % KOH (*filled circles*), 20 % KCl (*half-shaded triangles*), 15 % KNO_3_ (*empty stars*), 10 % K_2_SO_4_ (*diamonds*)

Based on voltammetry, the analysis of NCM capacity behavior was done in these electrolytes. The dependence of specific capacity of the NCM on potential scan rate is in Fig. 5. At a low scan rate, the top values of capacitance for NCM were gotten in 40 % KI electrolyte, which is connected with provided sufficient time to form EDL by К^+^ ions on the NCM surface and with Faraday reactions of iodine transfer on the carbon surface. While increasing the scan rate, the speed of charge spread is reduced within certain pores and the reduction of material capacity. A small amount of transportation pores are in the NCM (to 15 % from the total volume of pores), which provide free access for ions of electrolyte in the micropores, that is why a number of micropores is growing at a high scan rate, where the EDL cannot be fully formed, which is the reason why the reduction of the material capacity is at a high charge/discharge speed.

**Fig. 6:**
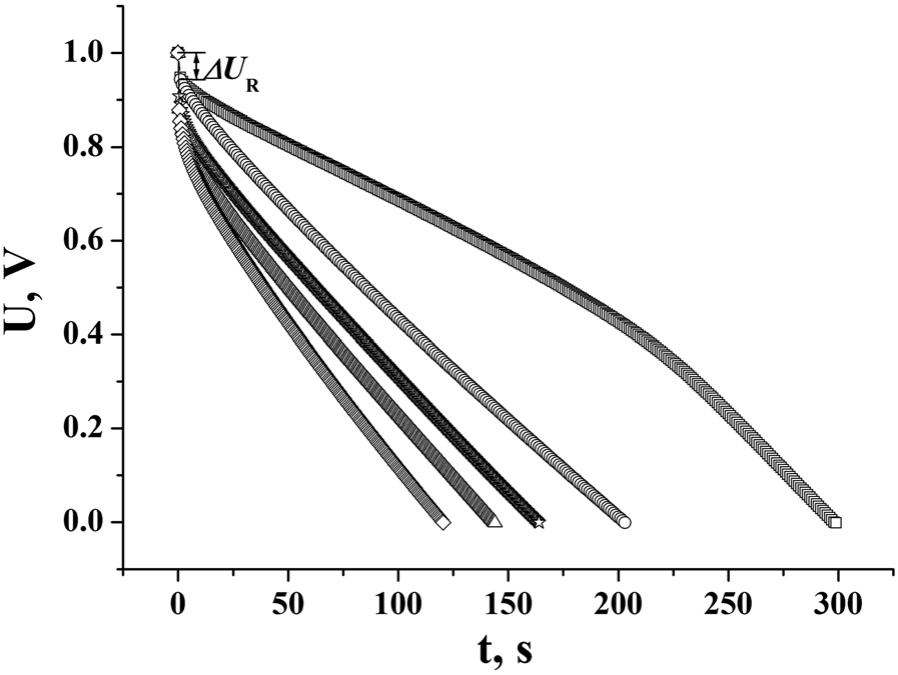
Discharge curves of EC, which were gotten at 50 mA current of discharge: 40 % KI (*empty squares*), 25 % KOH (*empty circles*), 20 % KCl (*triangles*), 15 % KNO_3_ (*empty stars*), 10 % K_2_SO_4_ (*diamonds*)

**Fig. 7:**
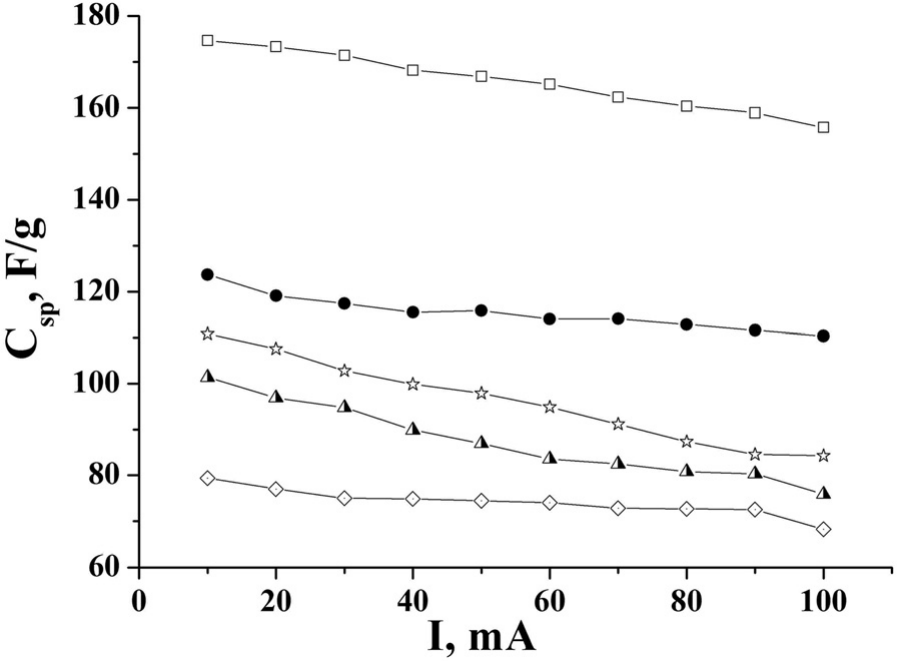
The dependence of NCM capacity on load current: 40 % KI (*empty squares*), 25 % KOH (*filled circles*), 20 % KCl (*half-shaded triangle*), 15 % KNO_3_ (*empty stars*), 10 % K_2_SO_4_ (*diamonds*)

Discharge capacity and internal resistance were calculated by galvanostatic method for the NCM in different electrolytes. The discharge curves of EC are show in Fig. 6. The dependence of capacity on discharge current for NCM in different electrolytes is shown in Fig. 7. As it is shown in the figure, the maximum capacity is observed for NCM/40 % KI electrochemical system, which is explained with high electrolyte conductivity (3168 × 10^4^ Ω^−1^ cm^−1^; [14]), and also simultaneous realization of two storage mechanisms: the formation of EDL by К^+^ ions on the carbon surface and the Faraday reactions of storage I^−^ on the carbon surface. Some lower, but stable, values of capacity were gotten for NCM in 25 % KOH. It can be explained, despite the high conductivity of 25 % KOH electrolyte (5403 × 10^4^ Ω^−1^ cm^−1^; [14]), that there is a single mechanism of storage in NCM/25 % KOH electrochemical system—the formation of EDL by К^+^ ions or by OH^−^ groups. Also, it must be noted that NCM capacity decreases in the sequence K_2_SO_4_ < KNO_3_ < KCl in 10 % K_2_SO_4_, 15 % KNO_3_, and 20 % KCl because the reduction of their conductivity of electrolyte is K_2_SO_4_ < KNO_3_ < KCl [14].

**Fig. 8:**
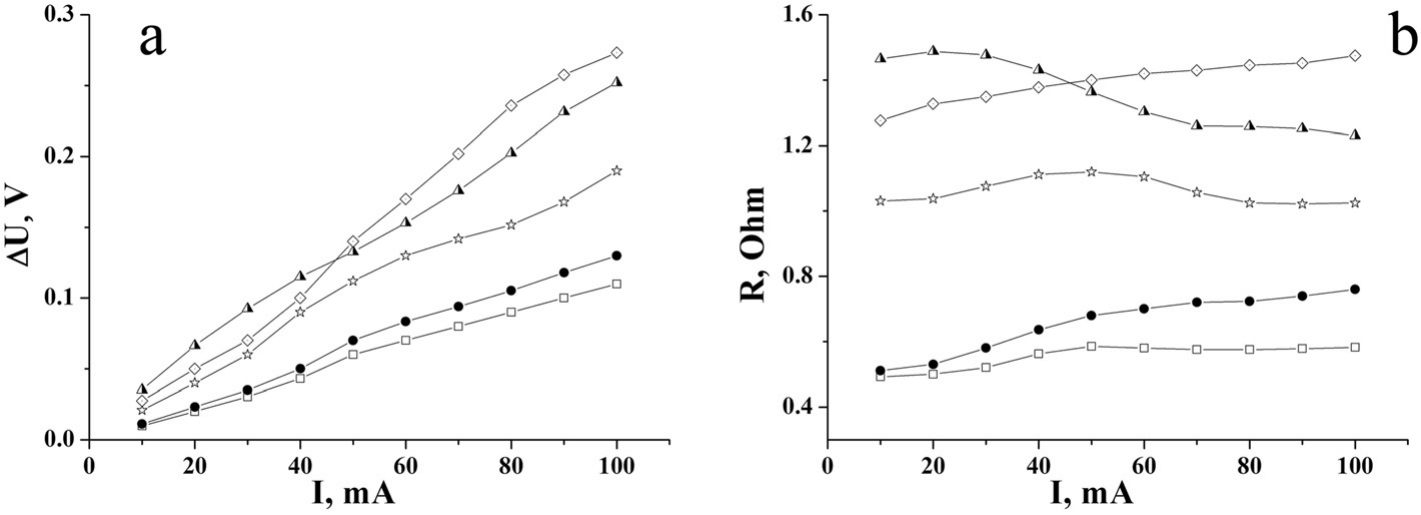
The dependence of voltage (**a**) and resistance (**b**) on load current in the К^+^-containing aqueous electrolytes: 40 % KI (*empty squares*), 25 % KOH (*filled circles*), 20 % KCl (*half-shaded triangles*), 15 % KNO_3_ (*empty stars*), 10 % K_2_SO_4_ (*diamonds*)

The voltage drop ***Δ****U*_R_ (Fig. 6) at constant current of charge/discharge shows the availability of Ohm resistance in NCM/electrolyte electrochemical system. In accordance to [15], the voltage drop ***Δ****U*_R_ *= IR* is determined like a point between voltage curve, which is lineal extrapolated, and instant time after closing the discharge circuit. If the voltage drop exceeds 20 % of maximum voltage, the current of discharge must be reduced to two, five, or ten times. The dependence of voltage drop and NCM resistance on load current is shown in Fig. 8. For every sample, the maximum operating current of discharge is at 100 mA, because after it increases, the voltage drop exceeds 20 % of the maximum voltage.

## Conclusions

According to potentiodynamic research, it was found that capacity of carbon materials depends on their electrochemically available surface which takes part in the formation of EDL. Besides the porous structure of carbon, conductivity of electrolyte is the equally important factor, which affects the value of capacitance and the total resistance of electrochemical system. It is established that the use of 40 % salt aqueous solution of KI is optimal.

It is shown that for NCM/40 % KI electrochemical system, the simultaneous realization of two mechanisms of energy storage are as follows: EDL formed by the К^+^ ions on the NCM surface and pseudocapacitive storage of I^−^ ions on the surface of the material that provides the capacity of NCM in the range of 175–155 F g^−1^ at discharging currents 10–100 mA.

It is found that EC based on NCM and 25 % KOH aqueous electrolyte is stable in all range of discharging currents and the material capacity approximately is 120 F g^−1^.

## Abbreviations

EC: electrochemical capacitors
EDL: electrical double layer
NCM: nanoporous carbon material
